# Supplementary material for: The interactome of intact mitochondria by cross-linking mass spectrometry provides evidence for coexisting respiratory supercomplexes
Source: Mol Cell Proteomics. 2017 Dec 8;17(2):216–32. doi: 10.1074/mcp.RA117.000470 (PMC5795388; doi:10.1074/mcp.RA117.000470)
Supplement: Supplemental Data [file supp_RA117.000470_133922_0_supp_23978_fzffwf.zip › spectra_annotation/mito_spectra_annotation/219-1-7-1-8-1.pdf]

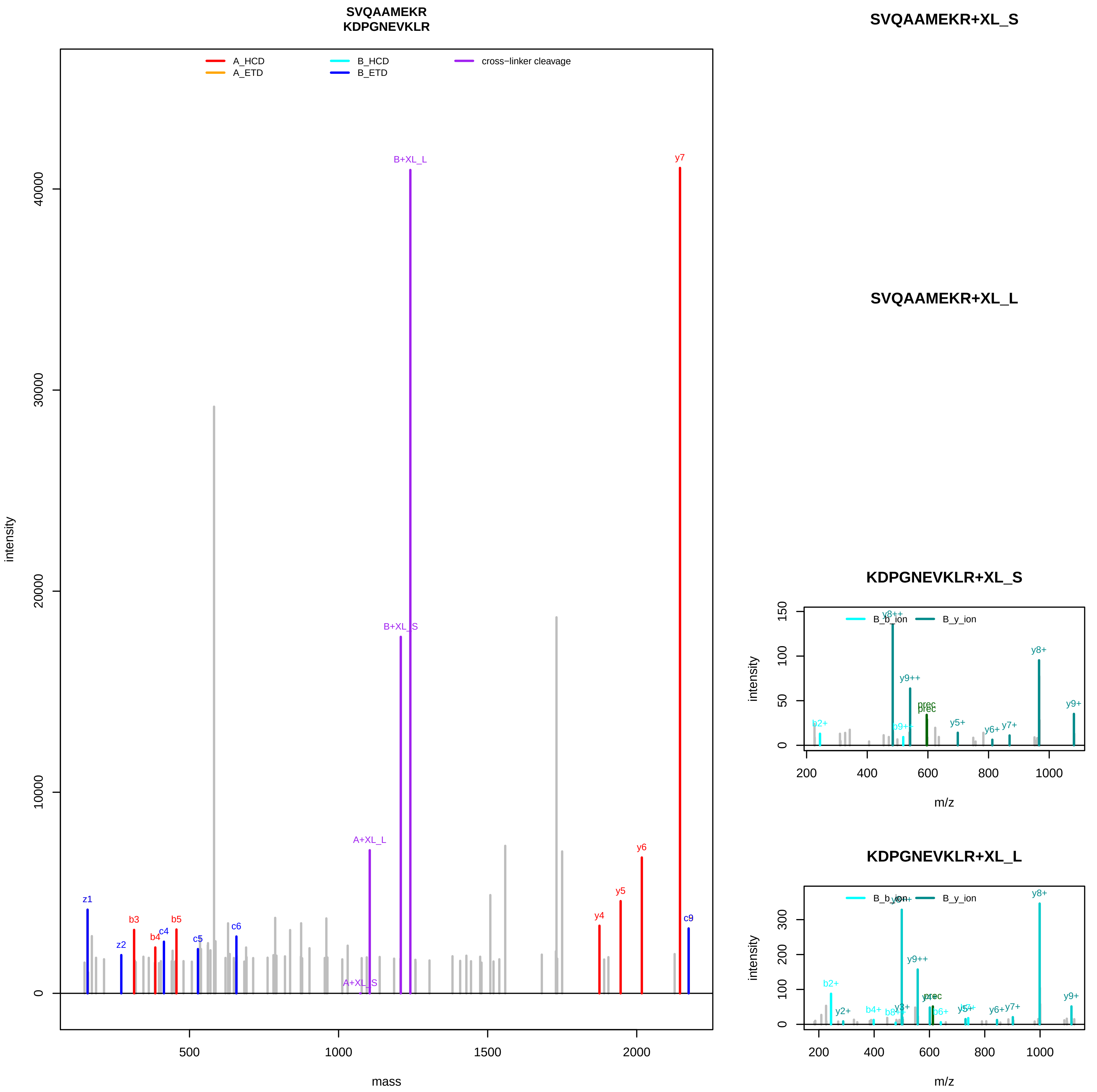

SVQAAMEKR
KDPGNEVKLR
A_HCD
A_ETD
B_HCD
B_ETD
cross−linker cleavage
intensity
mass
B+XL_L
y7
B+XL_S
A+XL_L
A+XL_S
y6
y5
y4
c9
z1
z2
b3
b4
c4
b5
c5
c6
SVQAAMEKR+XL_S
SVQAAMEKR+XL_L
KDPGNEVKLR+XL_S
KDPGNEVKLR+XL_L
B_b_ion
B_y_ion
m/z
y8++
y9++
y8+
y9+
prec
b2+
b9++
y5+
y6+
y7+
y2+
b4+
y3+
b8++
b6+
b7+
